# Factors associated with physical function in patients after surgery for soft tissue sarcoma in the thigh

**DOI:** 10.1186/s12891-023-06797-w

**Published:** 2023-08-18

**Authors:** Takuya Fukushima, Yusuke Okita, Noriko Watanabe, Shota Yokota, Jiro Nakano, Akira Kawai

**Affiliations:** 1 Department of Musculoskeletal Oncology and Rehabilitation, National Cancer Center Hospital, Tokyo, Japan; 2 Faculty of Rehabilitation, Kansai Medical University, Osaka, Japan

**Keywords:** Musculoskeletal Tumor Society score, Physical function, Quadriceps, Soft tissue sarcoma, Timed up and go test

## Abstract

**Purpose:**

This study aimed to examine the validity of the timed up and go test (TUGT), which is a representative, objective, and functional assessment that can evaluate walking speed, strength, and balance, and determine the significant factors associated with physical dysfunction in the early postoperative period in patients with soft tissue sarcomas (STSs).

**Methods:**

This retrospective, single-center, observational study conducted at the National Cancer Center Hospital included 54 patients with STSs in the thigh who underwent surgery. The Musculoskeletal Tumor Society (MSTS) score, which subjectively evaluates the affected limb, was evaluated at discharge, and TUGT was performed preoperatively and at discharge. Higher scores indicated good limb function in the MSTS score and poor performance in the TUGT. Spearman’s correlation analysis was performed to identify the relationship between the MSTS score and TUGT. A receiver operating characteristic curve was used to calculate the cut-off value of the change in pre- and postoperative TUGT for an MSTS score of ≥ 80%. To examine the significant factors associated with physical dysfunction, multivariate regression analysis was performed using the change in pre- and postoperative TUGT as the dependent variable.

**Results:**

Postoperative TUGT and the change in pre- and postoperative TUGT were significantly associated with the MSTS score. The cut-off value for the change in pre- and postoperative TUGT for acceptable affected lower-limb function was 3.7 s. Furthermore, quadriceps muscle resection was significantly associated with the change in pre- and postoperative TUGT in the early postoperative period.

**Conclusions:**

TUGT could be a useful objective evaluation tool for postoperative patients with STSs. The cut-off value for the change in TUGT can be used to monitor postoperative recovery. If recovery is prolonged, a rehabilitation program can be designed according to the severity of the functional impairment in muscle strength, balance, or gait. In addition, sufficient information should be obtained regarding the presence or absence of quadriceps resection, which has a significant impact on postoperative performance.

## Introduction

Soft tissue sarcomas (STSs) are rare heterogeneous neoplasms originating from mesenchymal cells, and more than half of STSs arise in the lower extremities [1, 2]. Limb-sparing surgery is the primary treatment for extremity STSs and adequate surgical margins are associated with improved survival [3]. However, surgery with wide and deep margins causes muscle and physical dysfunction owing to a decrease in muscle tissue [4, 5]. Furthermore, these impairments induce physical inactivity [6] and accelerate muscle and physical dysfunction. To evaluate the physical function, the Musculoskeletal Tumor Society (MSTS) scoring system was developed in 1985 and revised in 1993 [7]. This system allows for multifaceted functional evaluation, including pain, function, emotional acceptance, support, gait, and walking ability. The MSTS score is one of the most widely used measures that has been validated and found reliable, allowing it to be used as a functional evaluation tool [8, 9].

In contrast, a previous systematic review showed the need for objective functional evaluation after surgery for lower-extremity sarcoma [10]. This opinion seems to be supported by the difference between subjective and objective evaluations. One-year follow-up using the MSTS score showed that postoperative function in patients with lower-extremity STSs improved to preoperative baseline levels [8]. However, evaluations using objective functional assessments of balance, gait, and physical performance have shown that physical function in bone tumors and STSs is impaired even in the long-term postoperative period [11]. Therefore, an objective functional evaluation may capture physical dysfunction in more detail. Considering that physical function has not fully improved even in the long-term postoperative period, quantifying physical function in the early postoperative period may provide important information for interventional strategies to enhance recovery.

The timed up and go test (TUGT) is one of the representative objective functional assessments, evaluating walking speed, strength, and balance [12]. They are easy to conduct as they are simple and do not require special equipment. We hypothesized that if TUGT could be validated as an evaluation of physical function in patients with STSs, it would be a clinically applicable tool that could objectively evaluate impairment to which patients with STSs were susceptible in the early postoperative period. However, there are very few studies on objective functional evaluations of surgically treated patients with STSs [10]. Furthermore, to the best of our knowledge, no reports have examined the physical function of patients with STSs in the early postoperative period.

Hence, the primary purpose of this study was to examine the validity of TUGT as an objective functional evaluation in the early postoperative period in patients with STSs by comparing its results with the MSTS score. The second purpose was to identify the significant factors associated with physical dysfunction in the early postoperative period that may assist in identifying patients who need intervention and enhance recovery.

## Methods

### Study design and population

This retrospective, single-center, observational study was conducted at the National Cancer Center Hospital (NCCH). Ethical approval was obtained from the Institutional Review Board of the NCCH (approval number: 2017–336). All procedures were performed in accordance with the ethical standards of the responsible committee on human experimentation (institutional and national) and the Helsinki Declaration of 1964 and its later versions. Informed consent for inclusion in this study or an equivalent alternative was obtained from all the patients.

Patients with STSs in the thigh who underwent surgery between April 2019 and March 2021 were enrolled in this study. The inclusion criteria were as follows: 1) inpatients diagnosed with STSs in the thigh, 2) underwent surgery, 3) age ≥ 20 years, 4) independence in activities of daily living without the use of an assistive device, and 5) availability for evaluation. The exclusion criteria were as follows: 1) communication difficulties, 2) poor general status, and 3) inability to perform the evaluation.

### Measurements

General and clinical information (age, sex, histologic diagnosis, first or recurrent, preoperative treatment, operational information, and length of hospital stay) of the patients were collected from medical records. Preoperative treatment included chemotherapy, radiotherapy, and chemoradiotherapy, and was categorized as present or absent. Operational information included operation time, blood loss, resection, flap, and muscle and nerve operations. Regarding resection, we investigated the presence or absence of wide resection. Regarding muscle resection, patients were categorized according to the presence or absence of quadriceps or hamstring muscle resection. Partial resection was also performed. Femoral nerve palsy and sciatic nerve palsy were recorded.

The participants were evaluated for the MSTS score and TUGT. The MSTS score was evaluated at discharge and TUGT was evaluated preoperatively (preoperative TUGT) and at discharge (postoperative TUGT).

#### Musculoskeletal tumor society score

The MSTS scoring system is the most widely used evaluation and is based on an analysis of factors pertinent to the patient as a whole and those specific to the affected limb [7]. It contains six items: pain, function, emotional acceptance, use of external support, walking ability, and gait. Each of these items was assigned a value of 0–5 points, and the total scores were divided by the maximum possible number of points (30 points). Subsequently, the score was obtained by multiplying the calculated point value by 100. A score of ≥ 80% was considered acceptable [13].

#### Timed up and go test

TUGT is a representative, objective, and functional evaluation that can evaluate walking speed, strength, and balance [12]. This test required patients to stand up out of the chair, walk 3 m, turn around, walk back to the chair, and sit down. Patients were given the following instructions: “stand up on the word ‘go,’ walk to the tape, turn around, walk back to the chair, and sit down.” The timing of the test began with the word “go,” and ended when the participant was seated [14]. Measurements were performed twice, and the fastest time was recorded.

### Statistical analysis

Wilcoxon’s signed-rank test was used to compare pre- and postoperative TUGT. Spearman’s correlation analysis was performed to identify the relationship between MSTS score and postoperative TUGT, and MSTS score and the change in pre- and postoperative TUGT. A receiver operating characteristic (ROC) curve was used to calculate the cutoff value of the change in pre- and postoperative TUGT for an MSTS score of ≥ 80%. To examine the significant factors associated with physical dysfunction, multivariate regression analysis was performed using the change in pre- and postoperative TUGT as the dependent variable. Variables with significance in the univariate analysis (*P* < 0.05) were included in the multivariate model. Statistical significance was set at *P* < 0.05. Data are expressed as the median (interquartile range [IQR]) or number and percentage of participants. Data were analyzed using the IBM SPSS Statistics version 27.0 software (IBM Corp., Armonk, NY, USA).

## Results

### Patient characteristics

Of the 82 eligible patients with STSs in the thigh, 54 were included in this study. The patient demographics and clinical characteristics are described in Table 1. The median age was 60.0 (40.8 to 68.5) years, and there were 15 (27.7%) and 12 (22.2%) patients diagnosed with myxofibrosarcoma and liposarcoma, respectively. Of these patients, 43 (79.6%) underwent wide surgical resection, with 26 (48.1%), 14 (25.9%), and 3 (5.6%) undergoing quadricep muscle resection, hamstring muscle resection, and femoral nerve palsy, respectively. Wound complication was observed in two cases (3.7%). The median preoperative TUGT was 6.6 (6.0 to 8.2) s, and the duration of hospital stay after surgery was 14.0 (9.0 to 19.0) days.

**Table 1:** Demographic and clinical characteristics data

Variables	
Age, median (IQR), years	60.0 (41.0 to 69.0)
Sex, n (%)	
Male	29 (53.7%)
Female	25 (46.3%)
Histologic diagnosis, n (%)	
Myxofibrosarcoma	15 (27.7%)
Liposarcoma	12 (22.2%)
Spindle cell sarcoma	6 (11.1%)
Myxoid Liposarcoma	5 ( 9.3%)
Dedifferentiated liposarcoma	3 ( 5.6%)
Alveolar soft part sarcoma	2 ( 3.7%)
Synovial sarcoma	2 ( 3.7%)
Undifferentiated pleomorphic sarcoma	2 ( 3.7%)
Dermatofibrosarcoma protuberans	2 ( 3.7%)
Others	5 ( 9.3%)
Recurrence, n (%)	9 (16.7%)
Preoperative treatment, n (%)	
No	37 (68.5%)
Chemotherapy	7 (13.0%)
Radiotherapy	6 (11.1%)
Chemotherapy and radiotherapy	4 ( 7.4%)
Wide resection, n (%)	43 (79.6%)
Operative time, median (IQR), min	181.5 (100.0 to 260.3)
Blood loss, median (IQR), ml	55.0 (19.5 to 171.3)
Flap, n (%)	11 (20.4%)
Quadriceps muscle resection, n (%)	26 (48.1%)
Hamstring muscle resection, n (%)	14 (25.9%)
Femoral nerve palsy, n (%)	3 (5.6%)
Sciatic nerve palsy, n (%)	2 (3.7%)
Wound complication, n (%)	2 (3.7%)
Preoperative TUGT, median (IQR), s	6.6 (6.0 to 8.2)
Hospital stays after surgery, median (IQR), days	14.0 (9.0 to 19.0)

*IQR* Interquartile range

### Pre- and postoperative timed up and go test

**Fig. 1:**
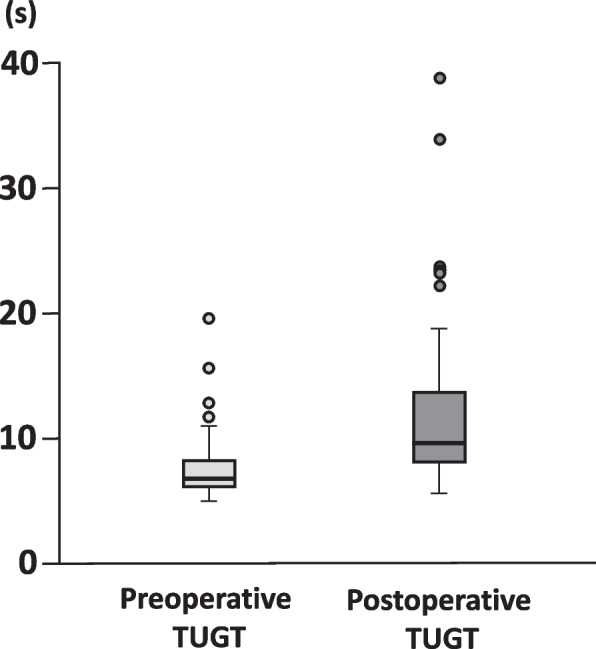
Pre- and postoperative timed up and go test

Preoperative and postoperative TUGT scores were 6.6 (6.0 to 8.2) s and 9.7 (8.1 to 13.7) s, respectively. The change in pre- and postoperative TUGT was 2.9 (1.1 to 6.5) s. In the Wilcoxon signed-rank test, a significant difference was observed between the pre- and postoperative TUGT (*P* < 0.001) (Fig. 1).

### Musculoskeletal tumor society score

**Fig. 2:**
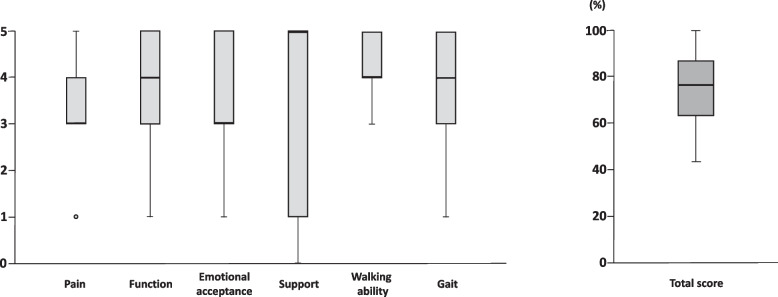
Musculoskeletal Tumor Society score

The medians of pain, function, emotional acceptance, use of any external support, walking ability, and gait were 3.0 (3.0 to 4.0), 4.0 (3.0 to 5.0), 3.0 (3.0 to 5.0), 5.0 (1.0 to 5.0), 4.0 (4.0 to 5.0), and 4.0 (3.0 to 5.0), respectively. The median total score was 76.7% (63.3 to 86.7) (Fig. 2).

### Association between the timed up and go test and the Musculoskeletal Tumor Society score

**Fig. 3:**
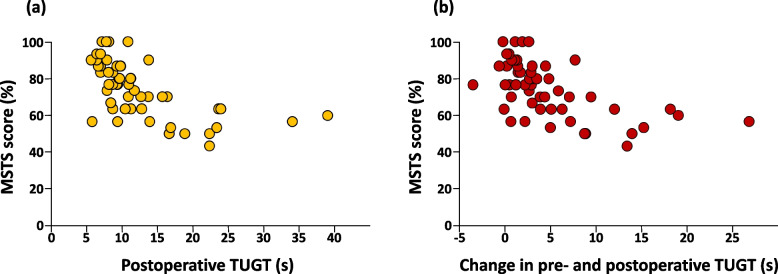
Relationship between Musculoskeletal Tumor Society score and timed-up-and-go test

**Fig. 4:**
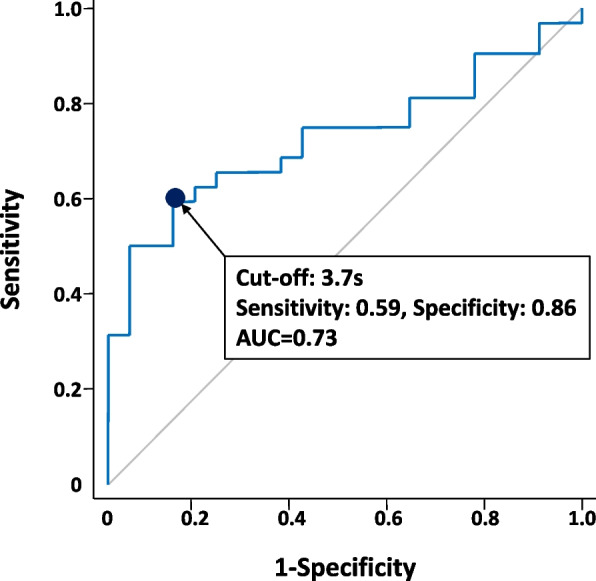
A receiver operating characteristic of change in pre- and postoperative timed up and go test for Musculoskeletal Tumor Society score (≥ 80%)

Spearman’s correlation analysis revealed a significant inverse association between postoperative TUGT and MSTS (*r* = -0.68, *P* < 0.001) and between TUGT and MSTS (*r* = -0.61, *P* < 0.001) (Fig. 3). The ROC analysis showed that the optimal cut-off value of change in pre- and postoperative TUGT for MSTS score was 3.7 s (sensitivity, 0.59; specificity, 0.86). The area under the curve value from the ROC analysis was 0.73 (Fig. 4).

### Factors associated with the change in pre- and postoperative timed up and go test

In the univariate regression analysis, wide resection (B = 3.78, β = 0.27, 95% confidence interval [CI] 0.02 to 7.55, *P* = 0.049) and quadricep muscle resection (B = 3.64, β = 0.32, 95% CI 0.66 to 6.62, *P* = 0.018) were identified as significant factors (*P* < 0.05). We included these variables in the multivariate regression analysis, which revealed that quadriceps muscle resection was significantly associated with changes in pre- and postoperative TUGT (B = 3.24, β = 0.29, 95% CI 0.15 to 6.33, *P* = 0.04) (Table 2).

**Table 2:** Factors associated with the change in pre- and postoperative timed up and go test

Parameters (reference)	Univariate analysis			Multivariate analysis		
	B (95%CI)	β	*P*-value	B (95%CI)	β	*P*-value
Age	0.05 (-0.04 to 0.15)	0.16	0.254			
Sex, male (female)	-0.76 (-3.91 to 2.39)	-0.07	0.631			
Recurrence, yes (no)	0.17 (-4.06 to 4.39)	0.01	0.937			
Preoperative treatment, yes (no)	2.47 (-0.85 to 5.79)	0.20	0.142			
Wide surgery resection, yes (no)	3.78 (0.02 to 7.55)	0.27	0.049	2.17 (-1.70 to 6.04)	0.15	0.265
Operative time	0.001 (-0.01 to 0.01)	0.02	0.901			
Blood loss	0.01 (-0.002 to 0.01)	0.20	0.148			
Flap, yes (no)	2.36 (-1.49 to 6.21)	0.17	0.225			
Quadriceps muscle resection, yes (no)	3.64 (0.66 to 6.62)	0.32	0.018	3.24 (0.15 to 6.33)	0.286	0.040
Hamstring muscle resection, yes (no)	-1.60 (-5.17 to 1.96)	-0.12	0.371			
Femoral nerve paralysis, yes (no)	-0.78 (-7.65 to 6.09)	-0.03	0.820			
Sciatic nerve palsy, yes (no)	2.74 (-5.56 to 11.04)	0.09	0.511			
Wound complication, yes (no)	-3.46 (-11.75 to 4.82)	-0.12	0.405			

In the multivariate analysis, it was adjusted with preoperative timed up and go test

*CI* Confidence interval

## Discussion

This study revealed that postoperative TUGT and the change in pre- and postoperative TUGT were significantly associated with MSTS scores, respectively. We also showed that the cut-off value of change in pre- and postoperative TUGT for acceptable affected lower-limb function (MSTS score ≥ 80%) was 3.7 s, and quadriceps muscle resection significantly associated with the change in pre- and postoperative TUGT in the early postoperative period in patients with STSs. To the best of our knowledge, this is the first study to examine the validity of TUGT as an objective functional evaluation and to reveal the significant factors associated with physical dysfunction in the early postoperative period in patients with STSs.

The MSTS score is a useful functional outcome and has been widely used in patients with STSs [7, 8]. On the other hand, the need for postoperative objective evaluation has also been presented [10]. In particular, the importance of balance and gait has been emphasized [10]. In response, we focused our attention on TUGT [12], which can assess balance, gait, and even muscle weakness that inevitably occurs in patients with lower-extremity STSs. Previous studies have shown that TUGT has been shown to assess physical function in a variety of patients with cancer [15] and has also been shown to objectively reflect the effectiveness of exercise therapy for patients with cancer [16]. In this study, postoperative TUGT and changes in pre- and postoperative TUGT were significantly associated with the MSTS score, the standard functional outcome. TUGT is thought to capture changes in physical function associated with surgery in the early postoperative period. Therefore, TUGT could be a useful objective evaluation for postoperative patients with STSs. It has been suggested that TUGT does not require tools or time to encourage its clinical application. In addition, the cutoff value of change in pre- and postoperative TUGT for an acceptable affected lower-limb function was clarified. Recovery in the early postoperative period can be positioned as a change of 3.7 s compared with the preoperative period, which is a useful indicator for proceeding with rehabilitation.

Quadriceps muscle resection was a significant independent factor associated with changes in pre- and postoperative TUGT. In other words, the results suggest that the knee extension mechanism has a significant effect. As mentioned previously, TUGT can assess strength, walking speed, and balance [12]. It is well known that the loss of muscle volume associated with quadricep resection leads to muscle weakness [17, 18]. Additionally, a decrease in the volume and strength of the quadriceps was associated with walking speed [19, 20]. Furthermore, the dynamic balance function has been shown to be significantly associated with quadriceps strength [21]. These previous studies support our results that quadricep strength affects TUGT performance. In patients with STSs, it is difficult to eliminate factors related to physical dysfunction in the early postoperative period, as revealed in this study, because the resection site is determined by the origin. This study suggests that if the quadriceps muscle is resected, rehabilitation should proceed with the expectation that postoperative recovery will be delayed. Furthermore, orthotics should be considered if the knee extension mechanism is significantly reduced. Thus, it would be useful to have sufficient information about the resected muscle during postoperative rehabilitation of patients with STSs.

This study had some limitations. First, this was a retrospective, observational study of patients from a single institution, which may have caused a patient selection bias. Second, the population is heterogeneous in terms of age, histological type, and various procedures required, including flap reconstruction. Third, tumor size could not be taken into account. This was compensated for by adjusting for clinically important factors such as extensive resection and the presence of muscle and nerve resection. Fourth, postoperative evaluations were performed at discharge and were not unified.

## Conclusions

Postoperative TUGT and changes in pre- and postoperative TUGT were significantly associated with the MSTS score. TUGT could be a useful objective evaluation tool for postoperative patients with STSs. The cut-off value of change in pre- and postoperative TUGT for acceptable affected lower-limb function (MSTS score ≥ 80%) was 3.7 s. This cut-off value can be used to monitor postoperative recovery. If recovery is prolonged, a rehabilitation program can be designed according to the severity of functional impairment in muscle strength, balance, or gait. Furthermore, quadriceps muscle resection was significantly associated with changes in pre- and postoperative TUGT in the early postoperative period. Therefore, it may be useful to proceed with the postoperative rehabilitation of STS patients using TUGT, an objective functional assessment, with sufficient information about the resected muscle.

## Data Availability

The data that supports the findings of this study are available on request from the corresponding author. The data are not publicly available due to privacy reasons.
